# Supplementary material for: A hierarchy of biomolecular proportional-integral-derivative feedback controllers for robust perfect adaptation and dynamic performance
Source: Nat Commun. 2022 Apr 19;13:2119. doi: 10.1038/s41467-022-29640-7 (PMC9018779; doi:10.1038/s41467-022-29640-7)
Supplement: Supplementary file 2 — Reporting Summary [file 41467_2022_29640_MOESM2_ESM.pdf]

nature research

Corresponding author(s): Professor Mustafa Khammash

Last updated by author(s): Mar 21, 2022

Reporting Summary

Nature Research wishes to improve the reproducibility of the work that we publish. This form provides structure for consistency and transparency in reporting. For further information on Nature Research policies, see our Editorial Policies and the Editorial Policy Checklist.

Statistics

For all statistical analyses, confirm that the following items are present in the figure legend, table legend, main text, or Methods section.

n/a	Confirmed	
☐	☒	The exact sample size (*n*) for each experimental group/condition, given as a discrete number and unit of measurement
☐	☒	A statement on whether measurements were taken from distinct samples or whether the same sample was measured repeatedly
☒	☐	The statistical test(s) used AND whether they are one- or two-sided<br>*Only common tests should be described solely by name; describe more complex techniques in the Methods section.*
☒	☐	A description of all covariates tested
☒	☐	A description of any assumptions or corrections, such as tests of normality and adjustment for multiple comparisons
☐	☒	A full description of the statistical parameters including central tendency (e.g. means) or other basic estimates (e.g. regression coefficient) AND variation (e.g. standard deviation) or associated estimates of uncertainty (e.g. confidence intervals)
☒	☐	For null hypothesis testing, the test statistic (e.g. F, t, r) with confidence intervals, effect sizes, degrees of freedom and P value noted<br>*Give P values as exact values whenever suitable.*
☒	☐	For Bayesian analysis, information on the choice of priors and Markov chain Monte Carlo settings
☒	☐	For hierarchical and complex designs, identification of the appropriate level for tests and full reporting of outcomes
☒	☐	Estimates of effect sizes (e.g. Cohen's d, Pearson's r), indicating how they were calculated

Our web collection on statistics for biologists contains articles on many of the points above.

Software and code

Policy information about availability of computer code

Data collection	1. MATLAB R2021a (academic use) -- Used for numerical simulations.<br>2. YouScope 2.0 (build: R2016-01, open source) -- Used for microscope and projector control in the cyberloop experiment (Figure 9(b)).<br>3. MATLAB R2016a (academic use) -- Used for cell segmentation, cell tracking, fluorescence quantification, and in silico simulation of biomolecular controllers in the cyberloop experiment (see Rullan et al. (2018) and Kumar et al. (2021) for more details).
Data analysis	All analyses in this study were performed on MATLAB R2018a and R2021a (academic use) platform using custom scripts. Manuscript figures were structured and formatted on Inkscape (v0.92, open source), MATLAB and TexStudio (v3.1.1, open source).<br><br>Simulation and manuscript figure generating codes are available here: https://doi.org/10.5281/zenodo.6373177

For manuscripts utilizing custom algorithms or software that are central to the research but not yet described in published literature, software must be made available to editors and reviewers. We strongly encourage code deposition in a community repository (e.g. GitHub). See the Nature Research guidelines for submitting code & software for further information.

Data

Policy information about availability of data

All manuscripts must include a data availability statement. This statement should provide the following information, where applicable:

- Accession codes, unique identifiers, or web links for publicly available datasets
- A list of figures that have associated raw data
- A description of any restrictions on data availability

The raw data (MATLAB .mat files) for the results of Figure 9(b) are available in the Source Data file. They are also available here: https://doi.org/10.5281/zenodo.6373177

Field-specific reporting

Please select the one below that is the best fit for your research. If you are not sure, read the appropriate sections before making your selection.

☒ Life sciences ☐ Behavioural & social sciences ☐ Ecological, evolutionary & environmental sciences

For a reference copy of the document with all sections, see nature.com/documents/nr-reporting-summary-flat.pdf

Life sciences study design

All studies must disclose on these points even when the disclosure is negative.

Sample size	For the experiments in this study, sample size refers to the number of cells tracked/stimulated for the full duration of the experiment. Two separate experiments were performed per controller to track in total 168 cells for the I-controller, 128 cells for the PI-controller and 178 cells for the PID-controller. Please refer to Kumar et al. (2021) for more details.
Data exclusions	There were few instances in which the media flow in the microfluidic chip got restricted due to uneven bonding between the chip and the glass slide. This resulted in media deficiency for the cells during the experiment. We have excluded data from those experiments in our final analysis. In addition to that, data from non-responding cells were manually removed from analysis and further consideration. These outliers (non-responding cells) constituted around 5-10% of total cells tracked throughout the experiment. Please refer to the Methods section for more details.
Replication	In the Cyberloop experiments, each cell is observed, controlled and targeted independently (segmented, tracked and quantified separately). Each cell has its own independent in silico controller which computes the input light intensity for the cell, and the DMD projection hardware targets only that particular cell with the computed blue light intensity.<br>On an average 79 (minimum 62) independent cells were targeted and tracked separately in each experiment. Experiments for each controller (I, PI and PID in Figure 9(b)) were performed twice on separate days, and similar results were obtained from both sets of experiments.
Randomization	Once cells were loaded into the microfluidic chip, one random pad with reasonable number of cells (in the microscopy field of view) was selected for the experiment. Only one experiment (with I, PI, or PID controller) was performed at a time. All cells within a fixed defined region in the microscopy field of view were considered for the experiment. This region was chosen based on cell density on the sample plane. As mentioned before, upper limit on number of tracked cells was set to be 140. This target region was chosen so that there were roughly that many cells inside the region. Cells were allowed to settle for 1 hour (without any stimulation) before starting the experiment.
Blinding	Researchers were not blinded during the study because the experiment routine, data processing and analysis were automated. Experiments were performed with cells randomly sampled from culture with no selection bias.

Reporting for specific materials, systems and methods

We require information from authors about some types of materials, experimental systems and methods used in many studies. Here, indicate whether each material, system or method listed is relevant to your study. If you are not sure if a list item applies to your research, read the appropriate section before selecting a response.

Materials & experimental systems

n/a	Involved in the study
☒	☐ Antibodies
☒	☐ Eukaryotic cell lines
☒	☐ Palaeontology and archaeology
☒	☐ Animals and other organisms
☒	☐ Human research participants
☒	☐ Clinical data
☒	☐ Dual use research of concern

Methods

n/a	Involved in the study
☒	☐ ChIP-seq
☒	☐ Flow cytometry
☒	☐ MRI-based neuroimaging
